# Homeostatic plasticity in neural development

**DOI:** 10.1186/s13064-018-0105-x

**Published:** 2018-06-01

**Authors:** Nai-Wen Tien, Daniel Kerschensteiner

**Affiliations:** 1 Department of Ophthalmology and Visual Sciences, Washington University School of Medicine, Saint Louis, USA; 2 Graduate Program in Neuroscience, Washington University School of Medicine, Saint Louis, USA; 3 Department of Neuroscience, Washington University School of Medicine, Saint Louis, USA; 4 Department of Biomedical Engineering, Washington University School of Medicine, Saint Louis, USA; 5 Hope Center for Neurological Disorders, Washington University School of Medicine, Saint Louis, MO 63110 USA

**Keywords:** Homeostatic plasticity, Neural development, Intrinsic excitability, Synaptic strength, Excitation/inhibition ratio, Patterned spontaneous activity

## Abstract

Throughout life, neural circuits change their connectivity, especially during development, when neurons frequently extend and retract dendrites and axons, and form and eliminate synapses. In spite of their changing connectivity, neural circuits maintain relatively constant activity levels. Neural circuits achieve functional stability by homeostatic plasticity, which equipoises intrinsic excitability and synaptic strength, balances network excitation and inhibition, and coordinates changes in circuit connectivity. Here, we review how diverse mechanisms of homeostatic plasticity stabilize activity in developing neural circuits.

## Background

**Fig. 1:**
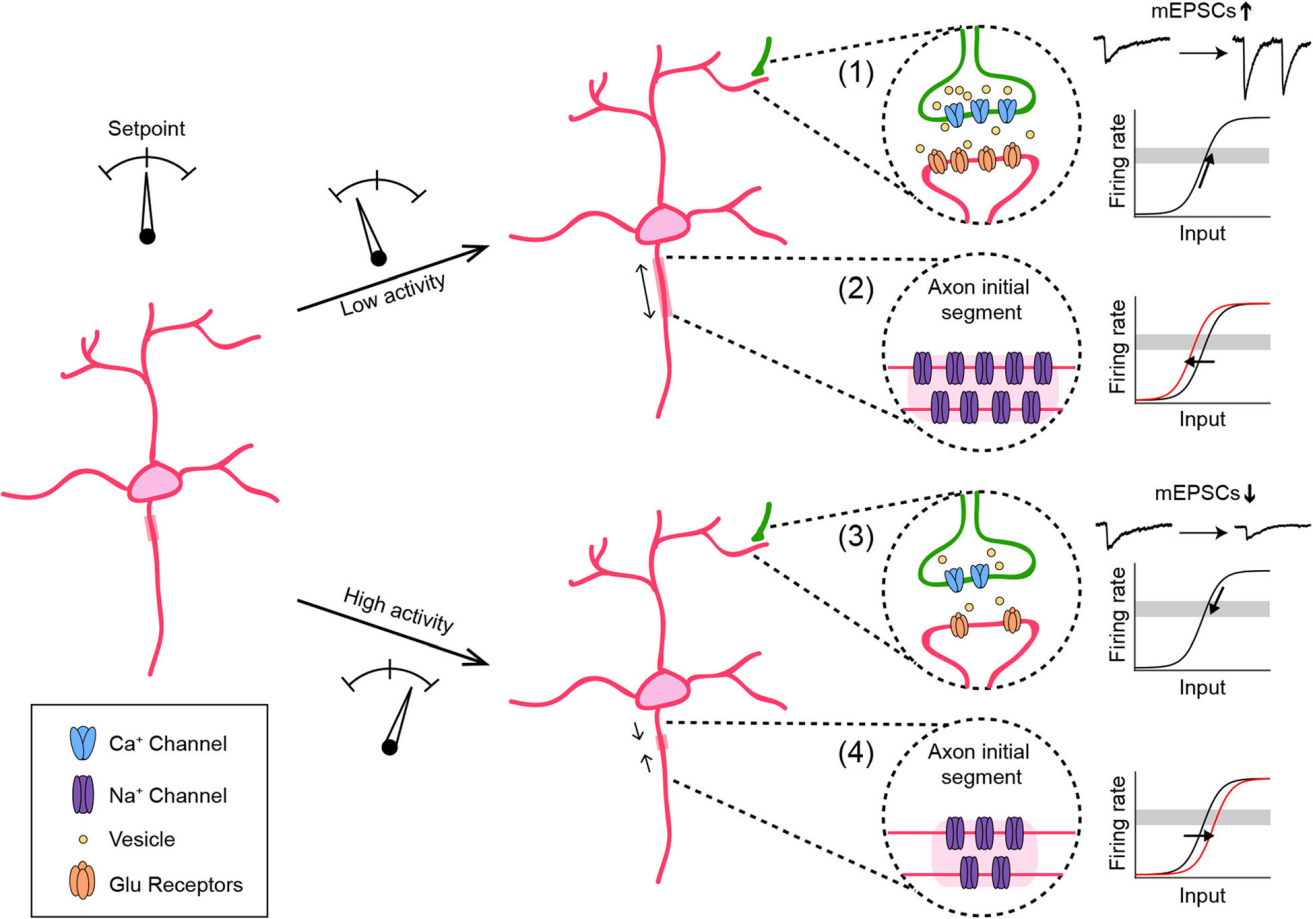
Diverse homeostatic plasticity mechanisms stabilize the activity of developing neurons. When the activity of individual neurons decreases below (1 and 2) or increases above (3 and 4) a setpoint, homeostatic regulation of synaptic strength (1 and 3) and/or intrinsic excitability (2 and 4) acts to restore normal activity. By increasing (1) or decreasing (3) synaptic input (e.g., changes in mEPSC amplitude or frequency), a neuron’s output firing rate can be shifted up or down to the target activity (grey area). By increasing (2) or decreasing (4) intrinsic excitability (e.g., changes in the length and location of AIS), a neuron’s input/output relationship can be modified

**Fig. 2:**
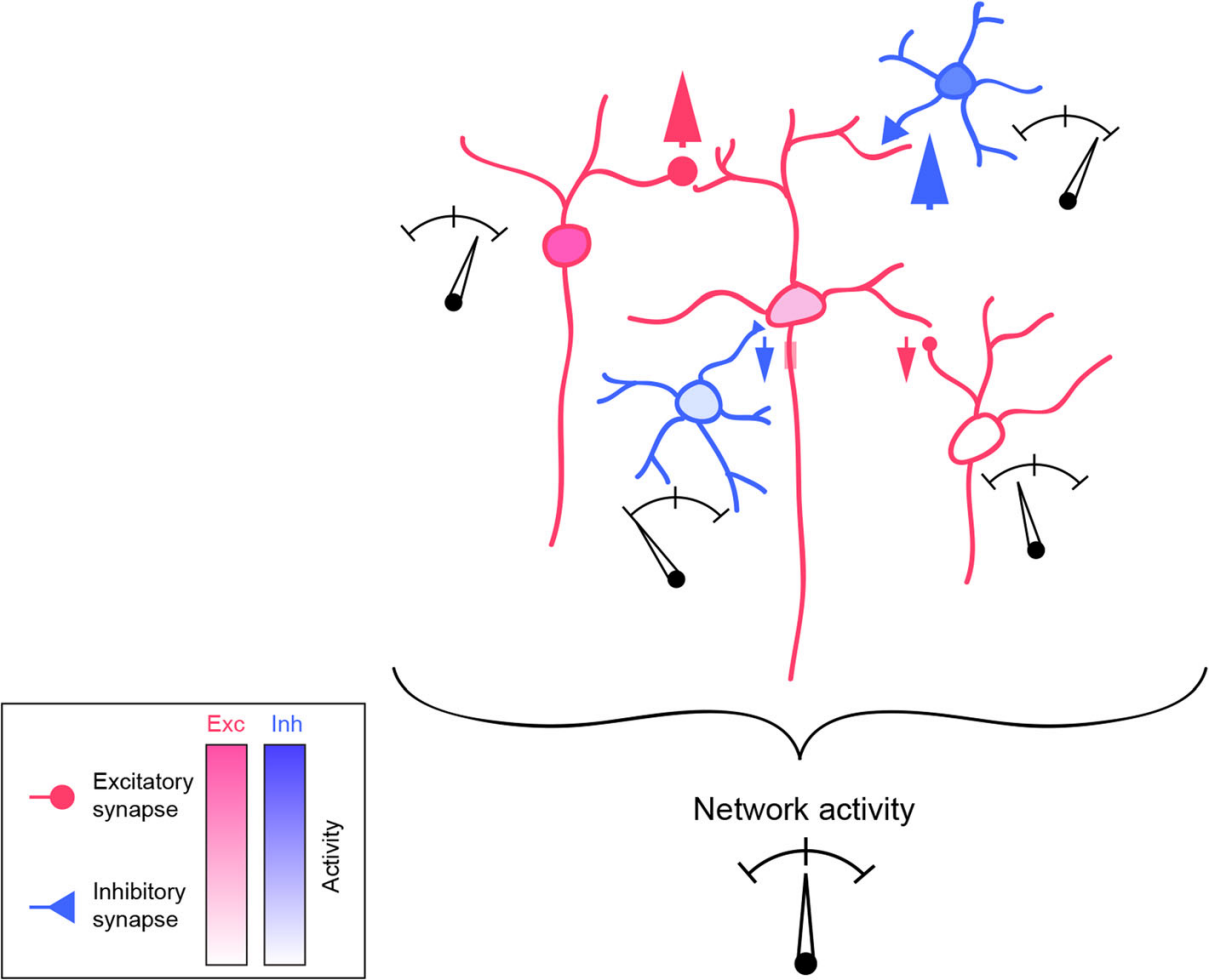
Network-level homeostatic plasticity stabilizes activity of developing circuits. Network activity homeostasis is achieved by balancing excitation (red) and inhibition (blue). Synaptic strength and connectivity can be regulated in a cell-type-specific manner to maintain network homeostasis. Upward/downward red arrows: increased/decreased excitatory drive; upward/downward blue arrows: increased/decreased inhibitory drive

Nervous systems face a constant challenge: how to maintain flexibility and stability at the same time. Neural circuits must stay flexible to allow for changes in connectivity and synaptic strength during development and learning. As changes in connectivity push neural circuits away from equilibrium, they need to maintain activity within a working range and avoid extremes of quiescence and saturation. Functional stability is maintained by homeostatic plasticity, which is defined broadly as a set of neuronal changes that restore activity to a setpoint following perturbation [1–3]. Recent studies have identified diverse homeostatic plasticity mechanisms triggered by a variety of perturbations. These mechanisms regulate dendritic and axonal connectivity of a neuron, as well as its intrinsic excitability (Fig. 1). In addition to maintaining the activity of individual neurons, homeostatic plasticity can act at a network level to coordinate changes in connectivity and excitability across multiple neurons to stabilize circuit function [4] (Fig. 2). Several recent reviews have covered the function of homeostatic plasticity in the mature nervous system [5–8]. Here, we focus on homeostatic plasticity in developing circuits.

### Homeostatic regulation of intrinsic excitability

Neuronal intrinsic excitability is determined by the density, distribution, and function of ion channels, and controls how synaptic inputs are converted into action potential outputs [9]. Several studies have found a reciprocal relationship between intrinsic excitability and synaptic inputs across development, which stabilizes activity [10–12]. As synaptic inputs increase in developing *Xenopus* retinotectal circuits, Na^+^ currents decrease, reducing intrinsic excitability [12]. Conversely, silencing synaptic inputs to developing *Xenopus* tectal neurons and *Drosophila* motorneurons increases Na^+^ currents and intrinsic excitability [10, 12, 13]. Several mechanisms mediate homeostatic changes in Na^+^ currents. Translational repression and post-translational phosphorylation reduce the density and open probability, respectively, of voltage-gated Na^+^ channels in *Drosophila* motorneurons and rat cortical neurons in response to elevated synaptic activity [11, 14–17].

Multiple ion channels in the same neuron can balance each other to stabilize activity [2, 18, 19]. For example, the A-type K^+^ channels *shal* and *shaker* are reciprocally regulated in motorneurons of *Drosophila* larvae: *shaker* is up-regulated in *shal* mutants, and *shal* is up-regulated in *shaker* mutants [20]. However, compensatory expression is not always a two-way street; in *Drosophila* mutants of the delayed rectifier K^+^ channel *shab*, increased expression of the Ca^2+^-dependent K^+^ channel *slo* prevents motorneuron hyperactivity, but, loss of *slo* does not increase expression of *shab* [21]. Neurons can synergistically regulate ion channels with opposite effects on excitability to restore activity. Silencing of pyramidal neurons cultured from visual cortex of rat pups with TTX increases Na^+^ currents and decreases K^+^ currents [22]. Finally, neurons of the same type with similar excitability can vary significantly in their membrane conductances, which may reflect the complex homeostatic interactions between ion channels [23–25] (for more discussion, see [26, 27]).

Detailed examination of the distribution of ion channels revealed an important role of the axon-initial-segment (AIS) in intrinsic homeostatic plasticity. Changes in length and location of the AIS, a specialized region with clusters of voltage-gated Na^+^ and K^+^ channels involved in spike generation, can counter the effects of sensory deprivation or photostimulation [28–31]. In mice, eye opening at postnatal day 13–14 shortens the AIS of pyramidal neurons in visual cortex [32, 33]. Together, adjustments in ion channel density, distribution, and function, resulting from changes in transcription, translation, post-translational modifications, and trafficking, can alter intrinsic excitability and balance changes in synaptic input to maintain activity homeostasis [9, 34–36].

### Homeostatic regulation of synapse strength and number

Homeostatic plasticity can regulate synaptic strength pre- and postsynaptically, and its dominant expression site can shift during development. In the early stages of network formation, miniature excitatory postsynaptic current (mEPSC) amplitudes increase when spike generation is blocked in cortical and hippocampal neuron cultures (i.e., suppression of intrinsic excitability), indicative of postsynaptic changes in AMPA receptor accumulation [37]. At later stages, presynaptic regulation of vesicle release and recycling is added, and mEPSC frequencies increase along with mEPSC amplitudes when spike generation is blocked [37, 38]. This suggests a developmental shift in the capacity for pre- and postsynaptic homeostatic plasticity [37]. Homeostatic control of synaptic strength has also been observed in vivo [39, 40]. The extent and expression site of this control depends on circuit maturation [41–45]. Homeostatic synaptic plasticity in layers 4 and 6 of primary visual cortex elicited by visual deprivation is restricted to an early critical period (postnatal day 16 to 21) [42, 43]. Later, homeostatic regulation of mEPSC amplitudes shifts to layers 2/3, where it persists into adulthood [42, 44]. The purpose of this shift in homeostatic plasticity across cortical layers remains unknown [41]. Chronic activity suppression by intracranial infusion of the Na^+^ channel blocker TTX or NMDA receptor blockers increases spine densities of developing thalamocortical neurons in the dorsolateral geniculate nucleus of cats and ferrets [46, 47]. Thus, homeostatic plasticity can regulate synapse number as well as strength [48–50].

In addition to homeostatic synaptic changes elicited by experimental perturbations, Desai et al. showed that across development, mEPSC amplitudes in layers 2/3 and 4 of rat primary visual cortex decrease as mEPSC frequencies and synapse numbers increase [42]. Retinogeniculate circuits provide another example of developmental homeostatic co-regulation [51–53]. Initially, many retinal ganglion cells converge onto thalamocortical cells, each forming weak connections. Then, for up to 3 weeks after eye opening, thalamocortical cells prune inputs, retaining synapses from fewer ganglion cells, which strengthen their connections [53, 54]. Thus, presynaptic neurotransmitter release, postsynaptic receptor abundance, and synapse number are homeostatically co-regulated during normal development and after activity perturbations. In several systems, the expression sites and the combination of mechanisms engaged shift across development [2, 3, 55–57].

### Homeostatic regulation of network activity

Homeostatic plasticity can stabilize the activity of individual neurons [54, 58, 59]. Neurons connect to each other in a cell-type-specific manner, forming circuits that perform specific functions. In the following sections, we discuss how homeostatic mechanisms are coordinated across neurons to stabilize circuit function [4, 60].

### Homeostatic regulation of network excitation and inhibition

Network activity is determined by the ratio of excitation and inhibition (E/I ratio) [1, 4, 61]. In response to perturbations, developing circuits can differentially adjust inhibitory and excitatory connectivity to alter the E/I ratio and restore activity [62–65]. In developing hippocampal and organotypic cerebellar cultures, TTX or glutamate receptor antagonists decrease inhibitory synapse densities and strengths, whereas blocking GABAergic transmission with bicuculline increases the density of inhibitory synapses. Similarly, brain slice recordings in barrel cortex layer 4 showed that sensory deprivation selectively reduces inhibitory input to layer 4 spiny neurons in young but not in adult animals [66, 67]. Activity-dependent changes in inhibitory synaptic transmission appear to be regulated non-cell autonomously, as activity suppression of individual presynaptic or postsynaptic cells failed to elicit compensatory changes observed after global application of TTX in neonatal cultured hippocampal neurons [65]. It has been suggested that inhibitory interneurons may sacrifice their own firing rate homeostasis to stabilize spiking of cortical pyramidal neurons after global activity blockade [4, 68]. Another example of network homeostasis comes from studies of monocular deprivation during the critical period [4]. Here, homeostatic plasticity adjusts recurrent and feedforward connections between layer 4 circuits and layer 2/3 circuits in primary visual cortex. Visual deprivation via intraocular TTX injection increases the excitatory drive and reduces inhibitory drive from layer 4 to layer 2/3, compensating for the lost excitatory sensory input [4, 69, 70]. Intriguingly, in another deprivation paradigm (i.e., lid suture), increased intrinsic excitability and decreased E/I ratios stabilize activity in layer 2/3, indicating the same circuit can use different combinations of homeostatic mechanisms to compensate for sensory deprivation.

In addition to regulating excitatory and inhibitory synapse strength and number, homeostatic plasticity can switch the transmitter phenotype of neurons from glutamate to GABA or vice versa to adjust the E/I ratio of developing circuits [71–73]. In the embryonic *Xenopus* spinal cord, the fractions of neurons expressing excitatory transmitters increase and decrease, respectively, when network activity is pharmacologically suppressed and enhanced. These switches in transmitter phenotype occur without changes in the expression of cell identity markers [74]. Similar to homeostatic regulation of inhibitory synapses, the activity-dependent transmitter switch is non-cell autonomous and depends on network activity, evidenced by the reciprocal relationship between the number of silenced cells and the ratio of neurons expressing GABA vs. glutamate [75]. Whether switches in transmitter phenotypes contribute to network homeostasis during normal development remains to be investigated [71].

### Homeostatic regulation of cell-type-specific connectivity

Recent advances in single-cell RNA sequencing together with large-scale morphological and functional surveys have revealed a great diversity of excitatory and inhibitory cell types, which serve distinct circuit functions [76–79]. This raises the questions whether, beyond categorical differences between excitatory and inhibitory neurons, homeostatic plasticity may act in a cell-type-specific manner to stabilize circuit function [80]. In the developing dentate gyrus, loss of excitatory drive by tetanus toxin expression results in reduced inhibitory input to granule cells [81]. This reduction is cell-type specific, affecting somatic innervation by parvalbumin-positive basket cells, but not dendritic innervation by calretinin- and somatostatin-expressing interneurons. Selective reduction of somatic inhibition efficiently restores the firing of granule cells [82, 83]. Similarly, monocular deprivation during a pre-critical period was shown to regulate feedback but not feedforward inhibition to layer 4 pyramidal cells in rat primary visual cortex [84]; and early hearing loss weakens inhibitory synapses from fast-spiking interneurons but not from low-threshold spiking interneurons onto pyramidal cells [85, 86].

Homeostatic regulation of excitatory connectivity can also be cell type specific [87]. In the developing mouse retina, following removal of their dominant B6 bipolar cell input, ONα retinal ganglion cells up-regulate connectivity with XBC, B7, and rod bipolar cells, but leave input from B8 bipolar cells unchanged. This cell-type-specific rewiring not only maintains the sustained activity of ONα retinal ganglion cells, but also precisely preserves their light responses. Thus, homeostatic plasticity can regulate inhibitory and excitatory connectivity in a cell-type-specific manner to maintain the activity and sensory function of developing circuits.

### Homeostatic regulation of patterned spontaneous activity

Throughout the nervous system, developing circuits spontaneously generate activity patterns that help refine their connectivity [88, 89]. Before eye opening, waves of activity originating in the retina propagate through the visual system and dominate activity up to primary visual cortex [90–92]. Retinal waves mature in three stages (I-III), in which different circuit mechanisms generate distinct activity patterns that serve specific functions in visual system refinement [88]. In mice, stage I waves, which are mediated by gap-junctional coupling of retinal ganglion cells, were first observed at embryonic day 17. Around birth, the wave generation switches to networks of cholinergic amacrine cells (stage II, postnatal day 1–10) followed in the second postnatal week by glutamatergic input from bipolar cells (stage III, postnatal day 10–14). The transitions between stages appear to be homeostatically regulated. When stage II (i.e., cholinergic) waves are disrupted by genetic deletion or pharmacological blockade of ß2 nicotinic acetylcholine receptors nAChRs, stage I waves persist until premature stage III waves take over [93–96]. Similarly, in VGluT1 knockout mice, in which stage III waves are abolished, stage II waves persist until eye opening [97]. Studies of developing spinal networks revealed an important role of excitatory GABAergic currents in homeostatic regulation of patterned spontaneous activity [98]. During development, GABA switches from excitatory to inhibitory as initially high intracellular Cl^−^ concentrations are lowered by the developmentally regulated expression of cation-chloride cotransporters [99, 100]. When spontaneous network activity in chick embryos was reduced by injection of a sodium channel blocker, excitatory GABAergic mEPSC amplitudes were found to increase because of an increased Cl^−^ driving force due to intracellular Cl^−^ accumulation [101, 102].

Although homeostatic mechanisms can restore spontaneous activity patterns following perturbations, the extent to which these activity patterns support normal circuit refinement varies depending on age and means of perturbation and needs to be further investigated [103–105].

## Conclusions

Developing circuits undergo profound changes in connectivity that threaten to destabilize their activity. Recent research has revealed a diverse set of homeostatic plasticity mechanisms, which safeguard activity of developing circuits. Different combinations of these mechanisms are recruited by different perturbations in different neuronal cell types at different stages of development. What signals control the recruitment of specific combinations of mechanisms is unclear and an interesting topic for future studies [41, 55].

Another important and mostly unanswered question is how activity setpoints are determined [2, 106–108]. Recent evidence suggests that this may occur during specific critical periods of development [109, 110]. Altering network activity in wild-type *Drosophila* during a critical period induces subsequent seizures, whereas correcting abnormal activity in mutant flies during the same period is sufficient to suppress seizures for life. Importantly, in the seizure-prone flies, homeostatic plasticity mechanisms are intact, but working toward the “wrong” setpoints. Insights into critical period timing and determinants of activity setpoints could have significant implications for the treatment of neurodevelopmental diseases including epilepsy and autisms [111–114].

## Abbreviations

AIS: Axon-initial-segment
E/I: Excitation/Inhibition
mEPSC: miniature excitatory postsynaptic current
